# Combining Body Mass and Shape Indices in Clinical Practice

**DOI:** 10.1155/2016/1526175

**Published:** 2016-02-29

**Authors:** Jesse C. Krakauer, Nir Y. Krakauer

**Affiliations:** ^1^Metro Detroit Diabetes and Endocrinology, Southfield, MI 48034, USA; ^2^City College of New York, 160 Convent Avenue, New York, NY 10031, USA

## Abstract

We present preliminary clinical experience with combined consideration of the commonly used BMI (body mass index) and the newly developed ABSI (a body shape index) using a point of care anthropometric calculator for comparisons of index values and associated relative risks to population normals. In a series of 282 patients, BMI and ABSI were close to being independently distributed, supporting the value of considering both indices. Three selected cases illustrate scenarios where assessment of ABSI together with BMI could inform patient care and counseling. These data suggest that combined assessment of BMI and ABSI may prove useful in clinical practice.

## 1. Introduction

Quantities derived from simple body measurements (anthropometrics), most commonly body mass index (BMI), have been extensively applied in population level risk assessment in recent years [1]. Despite the introduction of multiple new biochemical and genomic tests, the availability and low cost of height, weight, and waist circumference measurements will likely continue to be of interest for epidemiologic and clinical risk assessment. BMI was derived more than a century ago by Adolphe Quetelet to express body weight (*W*) independently of height (*H*) as BMI = *W* · *H* ^−2^ and relates to the historical discipline of allometry, with its focus on power-law scaling of biological quantities [2]. Concern regarding the appropriateness of health assessment based on BMI guidelines has arisen with recognition of the “obesity paradox,” perhaps better designated the “BMI paradox” [3]: a large cardiovascular and general medical and surgical literature finds the risk for adverse outcomes in many cohorts is actually lower for people whose BMI is modestly elevated (25–35, Overweight to Obesity I using World Health Organization (WHO) BMI range definitions [4, 5]) compared to people with WHO Normal BMI of 18.5–25.

The relationship of abdominal obesity to cardiovascular risk is well established. More specifically, there is evidence that metabolic risk correlates with the extent of visceral obesity, while subcutaneous fat is actually a source of protective adipokines. However, partitioning of abdominal obesity requires imaging technology not available to clinicians. The measurement of waist circumference (WC) has become the predominant indicator of abdominal obesity and associated visceral obesity, and WC cutoffs are suggested by national and international health organizations to supplement BMI for obesity assessment [5]. In fact, WC is strongly predictive of cardiovascular risk. However, there is a very high simple correlation between BMI and WC of *r* ≈ 0.7–0.9 [3, 5, 6]. Efforts to better isolate abnormal abdominal shape apart from BMI have included WC to hip circumference ratio and height to WC ratio. These demonstrate lower but still clinically important correlations with BMI, 0.4–0.5 [7]. Using these indices in conjunction with BMI to better assess risk is complicated by their correlations with BMI.

Several years ago, to address the above concerns, we introduced a body shape index (ABSI) based on an allometric power-law relationship between WC and BMI. ABSI was derived empirically from the National Health and Nutrition Examination Survey (NHANES) 1999–2004, a population sample of the United States [6]. The resulting ABSI was, as intended, almost independent of BMI, both for NHANES and, with minor adjustments, for studies from a number of other geographic regions [7, 8]. Further, we analyzed mortality follow-up data from NHANES and from a British study. In both, ABSI showed a direct association with mortality, with near log-linear risk increase, especially over the higher range of ABSI [6, 7]. Based on results from Cox proportional hazard modeling for mortality risk and Akaike information criterion score differences, we showed for these cohorts that ABSI predicted mortality better than BMI, WC, or waist/height and waist/hip ratios [7].

There are now dozens of studies that have evaluated the correlation of ABSI with various health-related measures and outcomes. We have previously listed and commented on some of them [1]. Relative risk (RR) estimates for ABSI have generally been similar in magnitude to those which can be made using BMI and other simple biometrics. In these studies, when significant, RRs are often modest, around 1.1–1.2 per standard deviation change in each indicator, and the RRs for different indicators are statistically similar to each other [9, 10]. However, few of the studies published to date exploited the statistical independence of BMI and ABSI to obtain combined RR values and confidence intervals, which we expect to be more informative than for the individual anthropometrics.

Much recent clinical application of simple biometrics has been based on combining BMI categories with WC cutoffs in an attempt to highlight the added risk of abdominal and visceral adiposity (corresponding to high WC). However, we demonstrated the limitations of WC cutoffs for risk stratification from national cohort data: WC cutoffs were exceeded, for example, 93% of the time for Obesity I (BMI 30–34.9) and 100% for Extreme Obesity (BMI > 40) individuals, so that the WC cutoff had little discriminatory power in these categories. On the other hand, ABSI was above average approximately 50% of the time across the entire range of BMI [1]. This motivates our hypothesis that adding ABSI to BMI in assessment could be valuable for clinical purposes beyond the prevailing BMI and WC cutoff-based categorizations, since the statistical independence of the two measures allows overall risk to be expressed more accurately as the product of the RR for BMI and RR for ABSI. To motivate future more definitive work, we present here preliminary results and case studies based on a practitioner's experience with point of care anthropometrics.

## 2. Materials and Methods

The first author practices general endocrinology in a single-specialty small group with an urban and suburban patient base. The office has electronic medical records, with visit documentation entered on a laptop taken into the exam room. Patients are weighed without shoes in light clothing on an electronic scale, and height is measured with a wall-mounted stadiometer. Height is recorded to the nearest 1/10 inch and weight to the nearest 1/10 pound. Patients have WC measured on a clinically selected basis. WC is determined with a nonrigid tape measure to the nearest 1/4 inch at the level of the iliac crests by a medical assistant following NIH guidelines [11].

Basic demographic data and height, weight, and WC were entered into an anthropometric calculator and the results are used in the course of the patient visit for diagnostic and educational purposes. Calculated quantities included ABSI, BMI, their *z* scores (number of standard deviations above or below the population average, controlling for age and sex), and RR relative to United States population means, including 95% confidence intervals for RR, as estimated from a mortality follow-up for a nationally representative cohort [6]. The ABSI and BMI attributable RRs are nonlinear empirically derived functions of the respective *z* scores. The combined RR is the product of the ABSI and BMI component RRs. A free version of our anthropometric calculator is available at https://nirkrakauer.net/sw/absi-calculator.html and can be experimented with to demonstrate how ABSI and BMI *z* scores and the estimated RRs and their confidence intervals depend on the entered height, weight, WC, age, and sex.

Over a one-year period, biometrics were recorded and analyzed for 291 patient visits. Nine patients had serial or duplicate entries, for 282 first-time individual analyses. Out of these, 3 selected cases are detailed below to illustrate scenarios where assessment of ABSI together with BMI could inform patient care and counseling.

## 3. Results

For the series of 282 patients, the average (±SD) age was 47 ± 15; 71% were female. The patient ethnicities were representative of our office practice, about 50% African American and 50% non-Hispanic White. The average BMI of the patients series was 36 ± 9 (1.3 ± 1.6 for *z* score, denoting the number of standard deviations above or below the NHANES 1999–2004 normals). For ABSI, the corresponding values were 0.0788 ± 0.0056 (−0.4 ± 1.2 for *z* score) (Table 1). The combined BMI-ABSI RR for mortality had higher standard deviation across the population than either BMI or ABSI attributable risks alone did (Table 1), suggesting that the combination enables better risk discrimination than either indicator used alone. Male patients had higher BMI *z* scores, but lower ABSI *z* scores, than females.

As expected from previous studies, there was relatively little correlation between ABSI and BMI, although it was statistically significantly different from zero (*r* = −0.21 using the raw values, −0.32 for the *z* scores; *p* < 0.001 for both). As Table 2 shows, the minority of patients presenting with below-average BMI (*z* < 0), who would thus be considered at low risk under the WHO obesity guidelines, were in fact just as likely to have above-average ABSI (*z* > 0), highlighting the potential of ABSI to identify patients at metabolic risk who may be missed by categorizations that are based primarily on BMI.

Three selected patient cases demonstrate the potential value of combined consideration of BMI and ABSI in the clinical setting.

### 3.1. Case 1: Low BMI with Comparatively Higher ABSI—Risk Reduction with Weight Regain and Stable WC

A 49-year-old woman was referred with 22-year history of type 2 diabetes mellitus. Maximum weight was 137 lbs 3 years previously. She had involuntarily lost to 106 lbs (BMI of 19, partly attributed to metformin diarrhea and terbinafine (for onychomycosis great toe) induced diarrhea and dysgeusia). Patient had retinopathy and years of no medical care or medication, which she attributed to being uninsured. Continuous glucose monitoring showed both nocturnal hyperglycemia and marked postprandial hyperglycemia. Biometrics showed below-normal ABSI (below average risk) but significantly elevated risk due to low BMI (based on the cohort data used to construct the calculator, the lowest-risk BMI is in the high normal to overweight range, with both lower and higher BMI incurring elevated risk).

Oral agents were discontinued and basal insulin was reinstituted, with subsequent addition of bolus insulin dosed with a calculating glucometer (ACCU-CHEK Aviva Expert). After three months, repeat anthropometrics showed weight gain to BMI of 21, with WC of 26 inches compared to 27 inches at initial assessment. The calculated BMI-ABSI combined RR decreased from above average (1.26) to just better than average (0.91).

In clinical practice, the adverse prognosis associated with low BMI may often be underappreciated. The decrease of risk with improvement in underweight is seen for BMI (RR 1.65 decreasing to 1.26 or 24%). Less intuitively, the stable WC as weight increased also reduced ABSI attributable risk (from 0.80 to 0.72 or 10%). Based on the statistical independence of RR-BMI and RR-ABSI, the combined risk decreased from 1.34 to 0.91, or 32%, demonstrating the complementary use of BMI and ABSI. The confidence intervals showed statistical significance for all the RRs, except for the final combined RR, which indicated now only average risk after the healthy weight regain.

### 3.2. Case 2: Normal BMI with Elevated ABSI—Risk Reduction by Relatively Greater Decrease in WC with Weight Loss

26-year-old woman was initially seen for postpartum hypothyroidism. Even with adequate thyroxine replacement and exercise and dieting efforts, she found it very hard to lose the 8 lb weight gained over prepregnancy. With normal-range BMI of 20 combined with relatively high WC of 33.75 inches (WHO cutoff is 34.6 inches for a woman), the WHO classification does not identify her at increased risk. However, her concerns were validated by a high ABSI and elevated combined RR. On the presumption, supported by the anthropometric risk indications, of a possible prediabetic metabolic disorder, metformin was prescribed and lifestyle measures redoubled. Repeat measurements after 5 months showed 12 lb weight loss and reduction in combined RR to significantly less than 1.

### 3.3. Case 3: Elevated BMI with Relatively Low ABSI—Overall Average Risk

This is a case of 60-year-old woman with lifelong weight struggles. Roux en Y bariatric surgery was performed 13 years ago at 248 lbs. Hypertension did not improve but is currently controlled on 5 medications. Creatinine has been stable at ≈2 mg%. A recent course of prednisone brought on 20 lbs weight gain. Anthropometrics showed near-extreme obesity using WHO criteria but normal combined RR thanks to relatively trim WC. Total body DXA% fat was normal (*z* score of +0.3). The patient was counseled that she not pursue aggressive medical weight loss and rather emphasize general health maintenance. In hindsight, although she was below her presurgical weight at 213 lbs (−35 lbs or −14% initial body weight), one might wonder how much she benefited health-wise from bariatric surgery.

Assuming that she were today a candidate considering bariatric surgery, her BMI of 39 and comorbid hypertension would be in favor of surgical intervention. However, on further assessment, her WC of 37 inches, while well above the accepted cutoff value, corresponded to a remarkably low ABSI *z* score of −3.15 (more than 3 standard deviations below the average value). One could therefore expect that abdominal obesity contributes little to her cardiometabolic risk. It is also notable that while the RR-BMI is elevated (1.17), RR-ABSI is below average (0.68), as is the combined RR (0.80). Based on the wide confidence intervals, all of which include 1, none of these is significantly different from average risk. Combined consideration of BMI-ABSI supported the clinical judgment that aggressive attempts at weight loss would not likely lead to better health outcome in this case.

## 4. Discussion

We presented experience with combination use of the anthropometrics BMI and ABSI to illustrate the potential advantages over BMI and WC cutoffs alone. The cases show situations where the currently recommended calculation and discussion of BMI as the premier clinical obesity indicator may result in an incomplete picture of a presenting patient's risk profile.

The measurement of WC beyond routine weight and height was feasible in the medical office setting, with minimal personnel training and equipment required. Dieticians and exercise physiologists could also readily use this modality.

We see at least three clear clinical benefits of our BMI-ABSI risk calculator:

1. On a population level, since ABSI quantifies body shape (particularly abdominal adiposity) in a manner that is independent of BMI (i.e., does not correlate with BMI), the ABSI-BMI combination provides better estimates of the relative risk of cardiometabolic disease and mortality.
2. Tracking changes in the relative risk over time from the calculator should help provide a way to evaluate the effectiveness of clinical interventions. Our finding that changes in mortality risk over time track changes in ABSI [7] supports this clinical application.
3. The BMI-ABSI risk calculator may be used to guide clinical decision-making and to assess comparative effectiveness. For example, ABSI might be an additional predictor of the likelihood of health benefits from bariatric surgery [12]. As another example, in the setting of medical weight loss, high ABSI may indicate a higher likelihood of response to metabolic versus appetite suppressive agents. Appropriate studies could verify the value of combined assessment of BMI and ABSI for particular medical conditions and in specific clinical settings, including hospital and managed care systems.

There are a number of additional directions we are exploring to make the risk calculations more useful, based on analysis of more large cohort studies with appropriate follow-up data. These include correlating with risk of metabolic syndrome and with conditions such as coronary artery disease, cancer, and stroke as well as with mortality; better understanding differences in risk associations across ethnicities and nations; and adding information from other body measures such as hip circumference to potentially improve risk assessment further.

## 5. Conclusion

In an era of increasing sophistication and cost of medical care, the simple measurements of height, weight, and waist circumference continue to have a role both for epidemiologists and medical practitioners and in fact offer prognostic value comparable to that of more expensive and invasive laboratory tests [13]. The combination of BMI and ABSI appears to outperform the now-routine use of BMI (or any other single index), while still fitting into the clinical setting, where basic measurements can be entered into calculators such as our online one.

## Figures and Tables

**Table 1:** Anthropometric quantities and calculated relative risks means and standard deviations.

	BMI	ABSI	*z*-BMI	*z*-ABSI	RR-BMI	RR-ABSI	RR-comb
All	36.2 ± 9.4	0.079 ± 0.006	1.36 ± 1.59	−0.35 ± 1.24	1.17 ± 0.29	1.00 ± 0.35	1.16 ± 0.43
Women	35.0 ± 8.9	0.078 ± 0.006	0.99 ± 1.31	−0.26 ± 1.21	1.12 ± 0.24	1.03 ± 0.36	1.13 ± 0.43
Men	39.4 ± 9.9	0.080 ± 0.005	2.16 ± 1.89	−0.58 ± 1.31	1.30 ± 0.35	0.95 ± 0.32	1.21 ± 0.44

**Table 2:** Contingency table for the distribution of patients with above- versus below-average BMI and ABSI. The chi-square statistic for this table is 1.02 (*p* = 0.31) indicating that above-average ABSI and above-average BMI were not significantly correlated.

	*z*-BMI > 0	*z*-ABSI < 0
*z*-ABSI > 0	94 (33%)	23 (8%)
*z*-ABSI < 0	146 (51%)	26 (9%)
